# Artificial intelligence–assisted smartphone thermal imaging for pedicled facial and head–neck flaps: An exploratory feasibility study using ΔT-based proxy perfusion grading

**DOI:** 10.1016/j.jpra.2026.10.016

**Published:** 2026-10-21

**Authors:** Fiedler LS, Veitinger A․, Leiser F, Ness N, Thiebes S, Sunyaev A

**Affiliations:** a Department of Otorhinolaryngology, Head and Neck Surgery, University Hospital Augsburg, Sauerbruchstraße 6, Augsburg, 86179, Germany; b Department of Otorhinolaryngology, University of Heidelberg, Heidelberg, Germany; c Department for Otorhinolaryngology, Head and Neck, Plastic Operations, SLK Kliniken, Heilbronn, Germany; d Chair of Information Infrastructures, School of Computation, Information and Technology, Technical University of Munich, Heilbronn, Germany; e Tongji University, Shanghai, China

**Keywords:** Thermal imaging, Artificial intelligence, Smartphone-based thermal imaging, Perfusion assessment, Pedicled skin flaps, Face, head and neck reconstruction

## Abstract

**Purpose:**

This pilot study evaluates the feasibility of artificial intelligence (AI)–assisted analysis of smartphone-based thermal imaging (SBTI) for perfusion assessment in pedicled facial, head and neck flaps. Because SBTI provides only false-color, non-radiometric thermal maps, ΔT-based grading relies on subjective human interpretation. We therefore investigate whether AI-derived perfusion probability maps were explored in relation to this manually generated proxy reference.

**Methods:**

Twenty-six patients undergoing reconstruction with pedicled skin flaps were examined with SBTI at four perioperative timepoints. ΔT-values were visually extracted from the FLIR color scale across predefined flap zones and translated into a five-grade perfusion score. A fine-tuned ResNet-50 model generated image-level probabilistic classification outputs regarding thermal similarity to patterns labeled as perfused versus non-perfused and uncertainty metrics. Analyses focused on T3 (end of surgery).

**Results:**

Thermal patterns varied substantially by flap type, thickness, and anatomical region. Random-pattern glabellar, nasal, and alar flaps showed physiologically lower surface temperatures and correspondingly reduced ΔT- and AI-derived prediction probabilities despite uneventful healing. No statistically significant correlation was observed between AI-derived perfusion probabilities and manually derived ΔT grades.

**Conclusion:**

AI-assisted SBTI is technically feasible but did not demonstrate meaningful quantitative agreement with manually derived ΔT-based grading in this small exploratory cohort. The findings primarily highlight the methodological limitations of false-color thermal interpretation and the need for radiometric imaging, validated perfusion references, and larger anatomically stratified datasets.

## Introduction

Reliable assessment of flap perfusion remains one of the most critical factors determining surgical success in reconstructive face, head and neck surgery. Pedicled and free skin flaps are essential tools for restoring function and aesthetics following tumor resection, trauma, or chronic wound treatment. However, early detection of perfusion compromise continues to depend largely on subjective clinical parameters such as color, capillary refill, and tissue temperature, which are operator-dependent and may fail to identify early ischemia. 1, 2, 3 The development of an objective, non-invasive, and reproducible method for perfusion assessment is therefore of high clinical relevance.

Thermal imaging (TI) enables visualization of cutaneous temperature patterns that indirectly reflect underlying blood flow, and has shown promise in several medical and surgical disciplines.^4^ The recent availability of smartphone-based thermal imaging (SBTI) systems has expanded the clinical applicability of this technique.^5^ SBTI offers a low-cost, portable, and contactless imaging modality for intraoperative and postoperative evaluation of skin flaps, allowing real-time monitoring without additional invasive devices.^6^^,^^7^ Prior studies have demonstrated its utility for perforator mapping, intraoperative decision-making, and postoperative flap surveillance in both pedicled and free flaps.

Despite these advantages, thermal image interpretation remains qualitative and user-dependent,^8^ limiting standardization across institutions.^9^^,^^10^ To overcome this limitation, our previous work^7^ proposed a structured, ΔT-based evaluation algorithm for SBTI in reconstructive surgery of the head and neck. By quantifying temperature differences (ΔT) across predefined flap zones, we previously demonstrated that ΔT values correlate with clinical perfusion assessment. However, ΔT-based grading itself remains an indirect and manually interpreted proxy measure. The present study therefore explores whether thermal image classifications provided by artificial intelligence (AI) demonstrate systematic distributional relationships with manually assigned ΔT-based categories. Our approach establishes SBTI as a reproducible and objective adjunct to clinical monitoring.^6^^,^^7^

AI provides new opportunities to provide a structured assessment of SBTI systems. AI models, specifically convolutional neural networks (CNNs) automate image processing and predictions and can improve diagnostic consistency.^11^ CNNs, as deep learning models trained on large image datasets, are capable of recognizing complex patterns that correspond to different perfusion states. Training CNNs on SBTI could enable standardized, real-time perfusion monitoring and early detection of vascular compromise during and after reconstructive procedures.

This study provides a first exploration of the technical feasibility of an AI–assisted analysis based on SBTI for pedicled flap assessment using manually derived ΔT-based grading as an exploratory proxy reference rather than a validated perfusion ground truth.

## Materials and methods

### Study course

Our study assessed the integration of AI models during thermal imaging for the assessment of patients undergoing reconstructive surgery for defects in the face and neck following tumor resections of varying extents. This investigation was prospectively conducted within the Department for Otorhinolaryngology/Head and Neck, Plastic Surgery at a tertiary care hospital, from November 2023 to June 2025. During the medical assessment, we collected relevant data such as age, gender, diagnosis, disease stage, diabetes mellitus status, smoking history (pack years), hypertension, anticoagulant use, history of previous surgeries in the operative area, details of the surgical procedure (including technique and date), location and size of the defect, intra-operative clinical assessment and Doppler signal, imaging details, postoperative complications, exact timing of post-operative assessments (T4), and the need for revision surgery, with follow-ups conducted for at least two months.

### Thermal imaging and manual false-color interpretation

SBTI and dynamic infrared thermography (DIRT), have now shown promise as an innovative tool in several medical specialties.^12^ Thermographic imaging detects long-wave infrared (LWIR) radiation, typically within wavelengths of approximately 8–14 µm, emitted by the body surface and translated into false-color temperature maps. Unlike visible light, which is perceived by the human eye, LWIR radiation is invisible and requires technology-based recording, analysis, and interpretation. By employing specialized cameras sensitive to LWIR wavelengths, such as infrared cameras (e.g. the FLIR One System), it becomes possible to capture and visualize LWIR radiation.^13^ Using the detection of infrared radiation emitted by the body, thermography produces accurate visual representations of surface temperatures, allowing areas of increased or decreased blood flow to be highlighted.^14^ Over these color-coded heat-emission differences perfusion patterns can be drawn.

Thermal imaging was performed using a smartphone-based infrared camera at four standardized perioperative timepoints (T1–T4). Because the device did not provide access to pixel-wise radiometric temperature data, all evaluations relied on manual interpretation of the false-color thermographic images provided by the native FLIR color map (see Table 1). For each image, three predefined regions were identified: the central flap zone (FZ1), the peripheral flap border (FZ2), and the surrounding non-flap tissue (FZ3). ΔT-values were visually estimated by comparing the predominant color shades within each region to the corresponding temperature intervals in the FLIR reference legend. Because the FLIR system provided only false-color visualization without access to pixel-wise radiometric temperature values, all ΔT estimates were approximate and dependent on manual interpretation of color gradients. Small shifts in palette scaling, ambient conditions, or image contrast may therefore substantially affect apparent ΔT estimations and downstream AI model predictions (Fig. 1, Fig. 2).

**Table 1:** Grading and temperature rainbow color coding in SBTI temperatures.

**Fig. 1:**
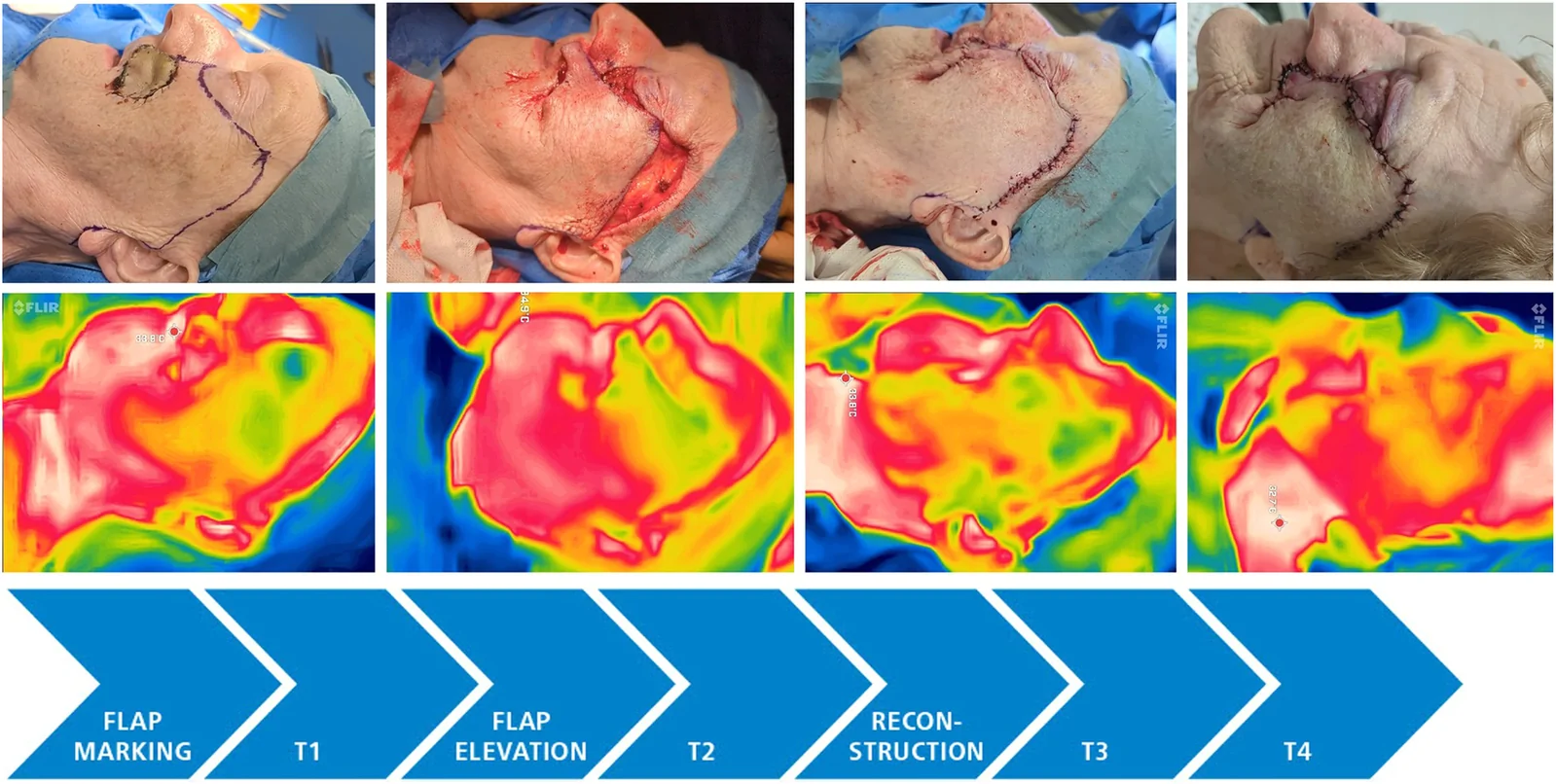
Modified Esser-flap for cheek reconstruction with timepoints T1-T4 in RGB and SBTI.

**Fig. 2:**
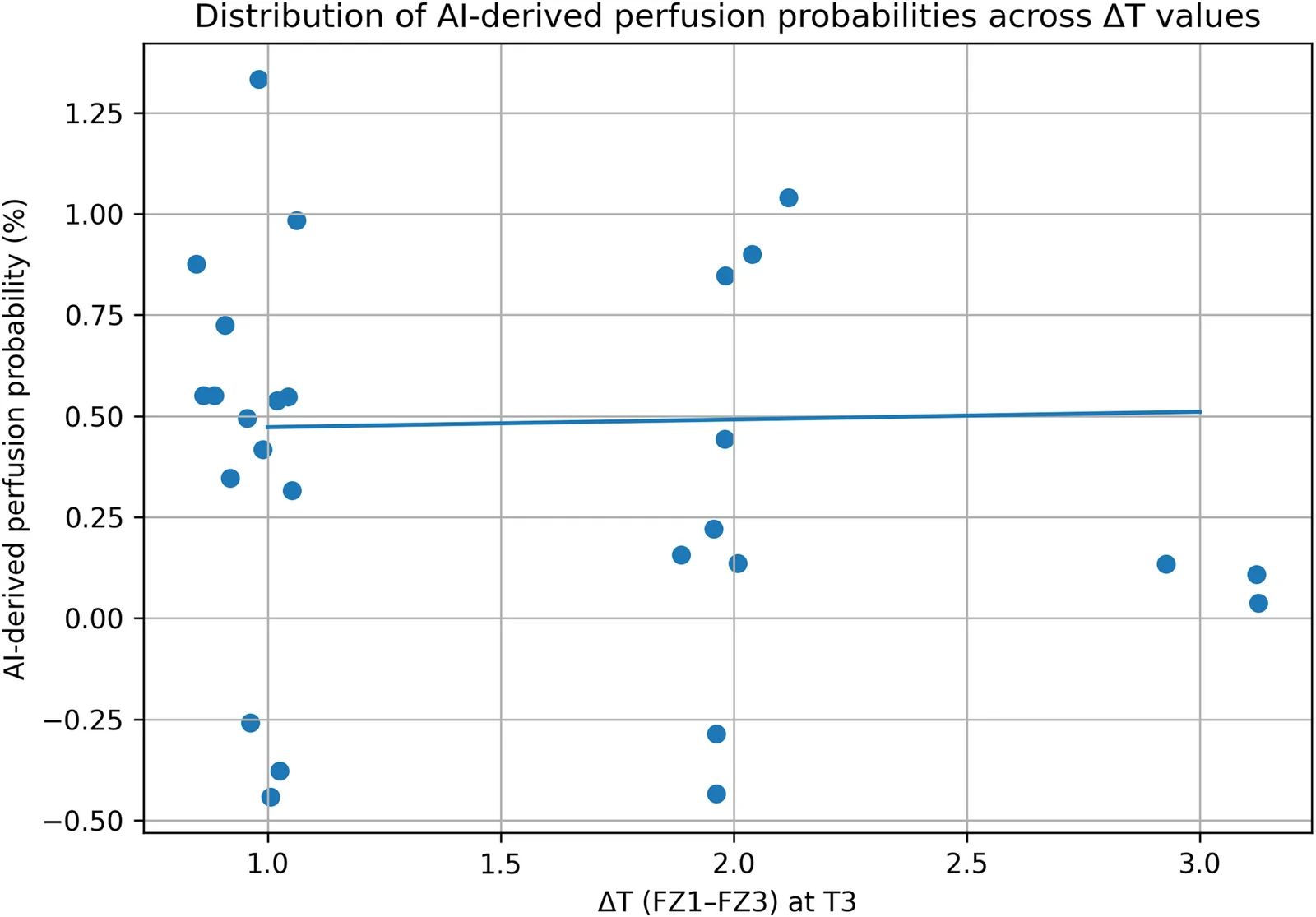
Scatter plot demonstrating the relationship between manually estimated ΔT (FZ1–FZ3) values at T3 and AI-derived perfusion probability scores. Jittered data points were used to improve visualization of overlapping observations. The solid line represents the linear regression fit.

### AI workflow and CNN model training

Providing an AI-assisted SBTI, we finetuned a CNN pre-trained on ImageNet on 68 RGB thermal images. The annotations of the images were provided by an experienced otorhinolaryngologist to ensure appropriate learning of the CNN. We split the images into train, test, and validation images following a 60-20-20 split. Each image was resized to 224 × 224 pixels for similar processing and normalized the images on the average values of ImageNet.^15^ To generate more images available for training, we augmented the training data using physiological plausible manipulations including random horizontal flip, rotation for up to ten degrees, and color variations representing incorrect measures or external temperature changes.

We chose to finetune a ResNet-50 model,^16^ pretrained on the ImageNet-1k data set to account for the limited dataset size, which prohibits robust training of a CNN from scratch. Only the final classification layers were retrained while leveraging pretrained feature extraction from ImageNet. Utilizing the pretrained ResNet-50 architecture, we added one dropout layer and a linear output layer using two neurons for a binary prediction. The provided images showed a predominance of perfused cases why the model was trained using a cross-entropy loss using balanced class weights.^17^ The retraining process was conducted using a batch size of 32, an Adam optimizer with a learning rate of 0.001 for a maximum of 25 epochs or if no improvement was achieved over 3 epochs.

The model did not perform pixel-wise segmentation or spatial perfusion mapping. Instead, the CNN classified the complete thermographic image as belonging to a perfused versus non-perfused pattern class based on the manually annotated training labels. The model generated image-wise predictions on the overall false-color image.

The front end is provided to the physician through a Flask-hosted website. The website allows the upload of images, which are then assessed through the best current model. After the inference, the website provides the prediction with the confidence (softmax value) and entropy. If the confidence is below a set threshold (we selected 0.70), the physician is encouraged to assess whether the prediction was correct or not. An active-learning-inspired feedback mechanism was implemented at the prototype level to explore future iterative model refinement. However, this component was not systematically evaluated within the present study and should therefore be considered conceptual only.

### Statistical analysis and evaluation

As reference standard, flap perfusion was graded using a predefined 5-grade ordinal scale derived from the manually estimated ΔT-values (see Table 1). Two surgeons independently assessed all thermal images, and discrepancies were resolved by consensus after joint discussion.

The AI-assisted SBTI was analyzed by examining the agreement between the AI-derived perfusion probabilities and the manually determined ΔT grades at T3, the most clinically relevant intraoperative time point. Statistical evaluation was descriptive, using Spearman correlation and median comparisons, acknowledging the subjectivity of manual false-color interpretation. All analyses adhered to strict patient-wise separation. Images from the same patient were never distributed across training, validation, and test subsets in order to minimize patient-level information leakage.

Because both ΔT grading and AI model predictions represented ordinal or probabilistic proxy measures rather than validated physiologic perfusion variables, analyses focused on exploratory distributional comparison rather than predictive performance evaluation. In addition to Spearman correlation and distributional comparisons of AI probabilities across ΔT categories were assessed descriptively.

## Results

### Patient cohort and flap types

A total of 26 patients undergoing reconstruction with pedicled skin flaps of the face, head and neck were included. The cohort ranged from 42 to 93 years of age. Flap sizes varied substantially (range 1 × 1 cm to 11 × 8 cm) and comprised bilobed flaps (n = 6), Rieger/Hatchet flaps (n = 6), nasolabial/Nelaton flaps (n = 5), frontotemporal or glabella advancement flaps (n = 3), cervicofacial/SCAI flaps (n = 4), and additional rotation-advancement designs (n = 2). All flaps demonstrated intraoperative clinical perfusion. Postoperative complications occurred in 1 out of 26 cases (3.8%), requiring revision surgery.

### Thermal characteristics across flap designs

Thermal distributions and ΔT patterns demonstrated clear design-specific profiles. Random-pattern flaps of the glabella and nasal dorsum (e.g., Rieger and glabella flaps, cases 44T12 × 1 and 67IIA04 × 5) regularly showed lower FZ1 temperatures, consistent with the thinness of the tissue, the longer diffusion-based perfusion pathways, and their tendency to cool rapidly after elevation. Despite low ΔT(T3) values and lower AI perfusion probabilities (AI perfusion probability score ∼33–42%), all these flaps healed uneventfully, underscoring that low thermal surface values in random-pattern flaps do not necessarily indicate compromised viability.

Nasal reconstructions, especially at the alar region, frequently appeared thermographically cooler due to the intrinsic temperature gradient of acral facial sites. This was particularly evident in patient 72T4503 × 2 (Nelaton flap), where a relatively low AI perfusion probability score (∼32–38%) occurred despite excellent clinical perfusion. These findings illustrate that anatomical location is a dominant confounder and limits direct comparability of absolute ΔT or AI-derived perfusion probability score across regions.

Large rotation or advancement flaps in the forehead and cervicofacial region (e.g., 82T304 × 5 or 82T3011 × 8) demonstrated wide thermal heterogeneity but uniformly successful healing, even where FZ1 cooling appeared more pronounced due to extended manipulation and larger surface areas. For these flaps, reduced AI perfusion probability score values (32–36%) reflected thermal dispersion rather than ischemia, again with intact postoperative outcomes.

### AI-Derived perfusion probabilities and ΔT relationship

AI-derived perfusion probability scores did not demonstrate statistically significant association or clinically meaningful agreement. Across the cohort, neither at T2 nor at T3 was a statistically significant correlation observed between ΔT(FZ1–FZ3) and AI perfusion probability score (Spearman ρ = 0.14, p = 0.47; Pearson r = 0.13, p = 0.51). Because no definitive ground truth for actual perfusion was available, performance metrics such as accuracy or sensitivity could not be calculated. Consistently, the model’s uncertainty measures (entropy, margin) also showed no stable association with elevated ΔT values or clinical outcomes.

### Clinical outcomes and alignment with thermal metrics

Healing was uneventful in 25 of 26 cases. One patient (75T103 × 2) with a 3 × 2 cm Nelaton flap to reconstruct the nasal ala developed peripheral dehiscence requiring surgical revision. This flap did not show extreme ΔT values or exhibit the lowest AI perfusion probability score. AI uncertainty values were mid-range and non-alarming. The flap type (Nelaton alar flap) is known to cool rapidly, reducing the predictive value of thermal patterns

## Discussion

This pilot study demonstrates the technical feasibility of applying convolutional neural networks to smartphone-based thermographic flap assessment. However, no statistically significant quantitative association was observed between AI-derived classification probabilities and manually assigned ΔT-based reference categories. The findings therefore should not be interpreted as validation of AI-derived perfusion assessment, but rather as an exploratory demonstration of workflow feasibility under substantial methodological constraints.

The heterogeneity of flap types and anatomical regions forms a second central factor influencing both thermal signatures and AI outputs. This heterogeneity substantially limits cross-case comparability and likely contributed to the absence of robust statistical association. Many facial, nasal, and perinasal pedicled flaps naturally cool more rapidly due to thin subcutaneous layers, random-pattern vascularization, or their location in acral zones characterized by physiologically lower surface temperatures.^7^ In this cohort, flaps from the glabella, nasal dorsum, and alar region displayed systematically lower absolute temperatures and reduced AI perfusion probability score values, yet healed without any clinical compromise. These cases show that low surface temperature does not uniformly indicate impaired perfusion and that absolute ΔT thresholds cannot be applied across flap categories without considering anatomical context. In this respect, the AI model behaved consistently: perfusion probabilities were lower in flap types that were thermally colder, reflecting the ΔT pattern, but without an associated shift toward a ‘non-perfused’ prediction or an interpretable risk signal. This highlights that the probabilistic outputs should not be viewed as absolute perfusion estimates; instead, they represent calibrated responses to the thermal features presented to the model.

The single postoperative complication in the series further reinforces the multifactorial nature of flap outcomes. The only patient requiring revision surgery presented neither extreme ΔT values nor conspicuous AI signatures during intraoperative measurements. This discordance suggests that the events leading to peripheral dehiscence were largely unrelated to macroperfusion, and more plausibly associated with mechanical factors or localized stress at the flap edges. Both manual ΔT interpretation and AI-assisted analysis, therefore, appear more suited to identifying global perfusion abnormalities than localized structural or mechanical risks, and neither modality should be viewed as an isolated predictor of postoperative success.

A key methodological and conceptual question arising from this study concerns the interpretation of AI-derived perfusion probability score. These values express the model’s probabilistic confidence regarding thermal similarity to patterns labeled as well-perfused, but they do not quantify physiological perfusion itself. Based on the heavy class imbalance in the training sample, the AI model did not sufficiently learn the specifics of postoperative complications. Future iterations of the AI model should take into account the considerably more severe costs of postoperative complication, increasing the penalties for these misclassifications. In addition, flaps with objectively intact microcirculation may display low AI perfusion probability score if they are anatomically predisposed to cooler surface temperatures. Conversely, even a high AI perfusion probability score does not guarantee flap stability when mechanical factors dominate postoperative risk.

The issue of class imbalance also warrants consideration. In a dataset dominated by clinically well-perfused flaps and very few pathological cases, any AI model is likely to overpredict normal perfusion patterns or demonstrate artificially low discriminatory capacity. While no naïve oversampling was applied in this project, future studies will need to consider patient-wise augmentation strategies that respect thermal physiology, avoid color-based distortions, and incorporate radiometric temperature data in order to reduce the inherent bias introduced by manually interpreted false-color images. Only with larger, prospectively collected datasets and patient-wise cross-validation can the true diagnostic potential of AI-assisted thermal interpretation be reliably assessed.

A central limitation of the present study is the absence of an objective physiologic perfusion ground truth such as indocyanine green angiography, Doppler flowmetry, or quantitative radiometric thermography. Consequently, both the ΔT-based reference grading and the CNN outputs represent indirect interpretative proxies rather than validated perfusion measurements. Because only a single postoperative complication occurred, meaningful predictive validation metrics such as sensitivity, specificity, AUROC, or complication prediction accuracy could not be robustly evaluated.

Overall, the findings of this pilot study underscore that while AI-assisted thermal image interpretation is feasible and produces clinically interpretable outputs, both the thermal data and the algorithmic predictions must be contextualized within flap design, tissue characteristics, and anatomical location. SBTI alone cannot replace clinical judgment, nor can AI probabilistic outputs be interpreted as quantitative perfusion metrics. Although no robust quantitative association was demonstrated in the present dataset, the technical feasibility of CNN-based thermographic interpretation suggests potential for future AI-assisted standardization once radiometric data, objective perfusion references, and larger datasets become available.

## Limitations

Several important limitations must be considered when interpreting the findings of this exploratory pilot study. First, no validated physiological perfusion ground truth was available. The ΔT-based grading system used as reference was derived from manual interpretation of false-color thermographic images rather than from objective modalities such as indocyanine green angiography, Doppler flowmetry, laser speckle imaging, or quantitative radiometric thermography. Consequently, both the manually assigned ΔT categories and the AI-derived outputs represent indirect proxy measures rather than validated perfusion parameters.

Second, the SBTI system provided only false-color visualization without access to raw radiometric temperature values. Small variations in color palette scaling, environmental conditions, image contrast, and subjective interpretation may therefore substantially influence estimated ΔT values and downstream CNN classification behavior. This limitation inherently restricts quantitative reproducibility.

Third, the dataset size was small and highly imbalanced, with only a single postoperative complication event. As a result, the CNN predominantly learned thermal patterns associated with clinically well-perfused flaps, limiting meaningful assessment of predictive performance, sensitivity, specificity, or complication detection. The AI model should therefore be regarded as exploratory and hypothesis-generating only.

Fourth, substantial clinical heterogeneity existed regarding flap type, tissue thickness, anatomical region, vascularization pattern, and flap dimensions. Facial and nasal flaps are known to demonstrate physiologically lower surface temperatures, which likely influenced both ΔT grading and AI-derived classification probabilities. This heterogeneity limits direct comparability across cases and may have obscured potential associations.

Fifth, the CNN performed image-level probabilistic classification rather than true pixel-wise perfusion segmentation. Accordingly, the AI-derived “perfusion probability” values should not be interpreted as direct physiological measurements of tissue blood flow, but rather as probabilistic similarity scores relative to the manually annotated training examples.

Finally, although an active-learning inspired feedback mechanism was technically implemented within the prototype system, this component was not systematically evaluated and therefore cannot be interpreted as evidence of improved model performance or adaptive clinical learning within the present study.

## Conclusion

This exploratory pilot study demonstrates the technical feasibility of AI-assisted interpretation of smartphone-based thermal images in pedicled facial and head-and-neck flaps. However, no meaningful quantitative agreement with manually derived ΔT-based reference grading was observed. Given the absence of validated perfusion ground truth, the small and imbalanced dataset, and substantial anatomical heterogeneity, the present findings should be interpreted as preliminary workflow feasibility only. Future studies require radiometric thermal imaging, objective multimodal perfusion references, anatomically stratified cohorts, and substantially larger datasets before clinically meaningful AI-derived perfusion assessment can be established.

## Funding

No external funding was received.

## Ethical approval

The study was approved by the Ethics Committee of the University of Heidelberg (Votum S-630/2023).

## Declaration of competing interest

The authors declare no conflicts of interest related to this study.
